# Exploring enablers and barriers to the use of chest compression feedback devices in advanced life support: a qualitative study

**DOI:** 10.1186/s13049-026-01607-3

**Published:** 2026-04-01

**Authors:** Øystein Myrlund Hansen, Charlotte Björk Ingul, Benjamin Stage Storm, Rita Solbakken

**Affiliations:** 1 Faculty of Nursing and Health Sciences, Nord University, Postbox 1490, 8049 Bodø, Norway; 2 Department of Medicine, Nordland Hospital Trust, 8092 Bodø, Norway; 3 Faculty of Medicine and Health Sciences, Department of Circulation and Medical Imaging, Norwegian University of Science and Technology (NTNU), Postbox 8905, 7491 Trondheim, Norway; 4 Faculty of Health Sciences, Department of Clinical Medicine, UiT – The Arctic University of Norway, Postbox 6050 Langnes, 9037 Tromsø, Norway; 5 Department of Surgery, Nordland Hospital Trust, 8092 Bodø, Norway

**Keywords:** Cardiopulmonary resuscitation, Chest compression feedback device, Cardiac arrest, Implementation science, Out-of-hospital cardiac arrest, Quality improvement, Qualitative research

## Abstract

**Background:**

In sudden cardiac arrest, high-quality chest compressions are crucial for survival with favorable neurological outcomes. Chest compression feedback devices improve chest compression quality and the likelihood of achieving return of spontaneous circulation. However, implementation remains challenging. Understanding enablers and barriers is essential to inform implementation strategies and device design.

**Methods:**

We conducted a qualitative exploratory study using semi-structured interviews with 15 healthcare professionals from pre- and in-hospital advanced life support settings in Norway and Denmark. Data were analyzed using reflexive thematic analysis to identify patterns of meaning related to enablers and barriers to device use.

**Results:**

Data analysis led to the construction of four themes:

(1) *The CPR sensor—a key physical interaction point,* where usability was shaped by ergonomics, as well as challenges related to sensor positioning and detachment. The sensor created a physical buffer that reduced the discomfort of feeling rib and sternum fractures and skin damage during compressions, helping providers maintain focus and perform compressions consistently.

(2) *Feedback—Device-to-rescuer communication,* where visual feedback was preferred and considered informative, while audio feedback was often seen as disruptive and difficult to perceive during compressions.

(3) *Organizing cardiac arrest treatment,* where team structure, leadership roles and protocol integration influenced device use. Clear assignment of responsibility and predefined equipment layout supported consistent use.

(4) *Perceived usefulness—an important enabling factor,* where feedback was especially valued in supervisory roles, supporting clinical oversight and decision-making, leading to device integration into team workflows. In the pre-hospital setting, the device supported effective guidance for bystanders. The device’s inability to provide physiological feedback raised questions about its future relevance.

**Conclusions:**

Successful implementation of chest compression feedback devices in advanced life support depends on user-centered design, role-sensitive feedback, and integration into clinical protocols. Key enablers included sensor ergonomics, visual feedback, and structured team roles, while barriers such as sensor detachment and disruptive audio feedback hindered sustained use. Future development should focus on multimodal feedback tailored to team functions and support physiology-guided resuscitation.

**Supplementary Information:**

The online version contains supplementary material available at 10.1186/s13049-026-01607-3.

## Background

Sudden cardiac arrest is a major global health problem and the third leading cause of death in Europe [1, 2]. High-quality chest compressions during cardiopulmonary resuscitation (CPR) are crucial for survival with favorable neurological outcomes [2, 3] and are described as a top priority in advanced life support (ALS) guidelines [4]. Mechanical chest compression devices are widely used in both in- and pre-hospital ALS systems; however, they have not been shown to be superior to manual chest compressions [4, 5]. Furthermore, some studies indicate a higher risk of iatrogenic chest injuries and worse neurological outcomes for patients resuscitated with mechanical compressions [6]. Manual chest compressions remain the recommended method according to current resuscitation guidelines [4, 5]. Given these limitations, this study focuses on manual chest compressions, and the use of chest compression feedback devices (CCFD), as improving manual compression quality remains a priority.

Performing high-quality chest compressions may be physically, cognitively, and emotionally challenging [7–9]. Subjectively assessing chest compression quality represents a significant challenge [10], which may partly explain why healthcare personnel often struggle to consistently provide high-quality chest compressions during the resuscitation attempt [11, 12].

The American Heart Association recommends the use of CCFD to improve CPR quality by providing real-time feedback on chest compression performance [2]. These devices offer objective feedback on key CPR metrics such as compression depth, rate, and recoil [2, 3, 13], which have been shown to improve chest compression quality [12, 14, 15] and increase the likelihood of return of spontaneous circulation [14, 16, 17].

Despite these benefits, several studies report challenges in implementing CCFDs in clinical practice [16, 18, 19]. Clinicians find the device not always necessary and it might be forgotten during resuscitation [18]. Interventions such as intravenous access and intubation are often prioritized over device placement [15, 19]. In addition, achieving correct positioning of the CPR sensor on the patient’s chest may be challenging [20], and the sensor may cause discomfort of the hands and wrists during compressions [19, 21, 22]. Using real-time feedback may increase chest compression providers’ perceived workload and contribute to fatigue [9, 22], while potentially reducing cognitive load for the rest of the resuscitation team [9].

Two studies from Germany and Finland have investigated clinicians’ acceptance of CCFDs and their associated user needs [23, 24]. Acceptance was primarily linked to familiarity with the device [23] and performance expectancy, including its perceived impact on patient-centered outcomes. Feedback should be intuitive and be easy to follow, and the study participants expressed strong support for the use of CCFDs [23, 24]. These findings contrast with two other studies: one in which Finnish emergency physicians rarely used the device [18] and another in which CCFDs were used in fewer than 25% of out-of-hospital cardiac arrests in Germany, despite being available. In both cases, the authors described the usage as low and infrequent [16].

Previous studies have demonstrated how CCFDs improve patient-centered outcomes [12, 14–17, 25, 26] and describes favorable attitudes toward the device [23, 24]. Yet, their adoption in real-world settings is limited [16, 18, 19]. In the Norwegian cardiac arrest trial “RescueDoppler” [27], we faced practical challenges in ensuring consistent use of the device by clinicians. This sparked our interest in exploring the underlying reasons for the varying use in clinical practice. Existing research on enablers and barriers to the use of CCFDs in ALS is scarce and predominantly based on quantitative methodologies [18, 19, 22, 23]. Several studies have examined the clinical effect of CCFDs [12, 14, 15, 17, 26], including two from Scandinavian settings [12, 15]. However, the practical and organizational factors that shape the implementation and use of CCFDs in real-world ALS settings are still largely unexplored, and recognized as a knowledge gap by the International Liaison Committee on Resuscitation [25].

The aim of this study is to explore and describe enablers and barriers to the use of CCFDs in ALS. The results may inform future implementation and device design.

## Methods

### Design

This study employed a qualitative exploratory design, using semi-structured in-depth interviews to explore and describe enablers and barriers to the use of CCFDs in ALS. This approach was chosen in accordance with qualitative research principles, and was considered suitable for exploring under-researched phenomena and capturing complex experiences [28]. The study was informed by a pragmatic philosophical framework. It adopted an epistemological and ontological stance in which knowledge is understood to be actively constructed through inquiry into lived experience [29]. The article is reported in accordance with the COnsolidated criteria for REporting Qualitative research (COREQ) checklist [30] (Additional file 1).

### Study setting

**Fig. 1:**
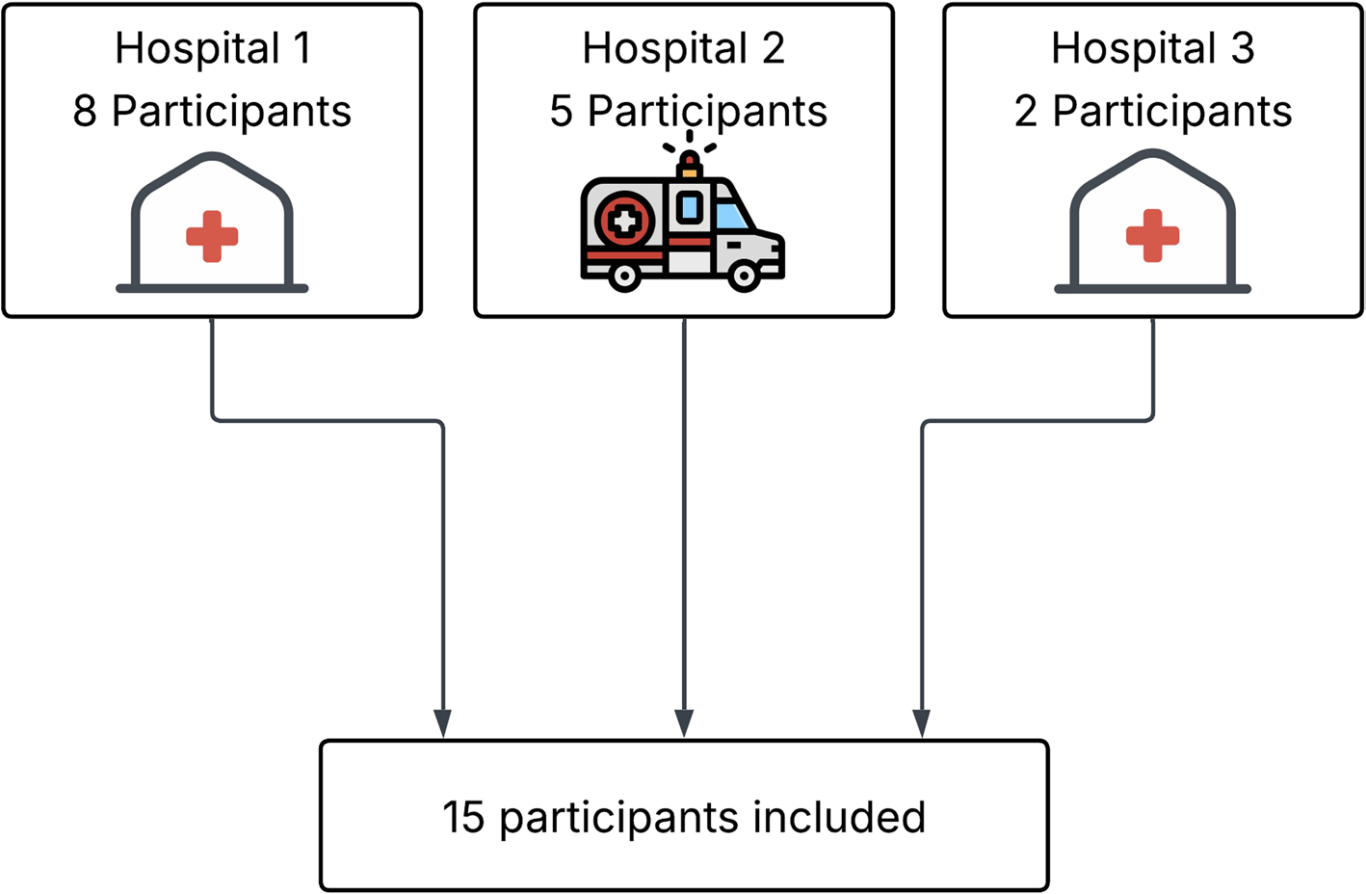
Overview of the recruitment process

To maximize findings, we chose to explore healthcare personnel’s experiences with CCFDs in both in-hospital and pre-hospital settings, thereby capturing a broad range of perspectives [28]. This study initially sought to focus on Norwegian clinical settings. However, as no eligible Norwegian pre-hospital services had implemented CCFDs, we extended the setting to include the pre-hospital service of one Danish hospital (Fig. 1).

### Recruitment

Inclusion criteria were limited to healthcare personnel with experience using CCFDs during ALS. Maximum variation sampling was employed to capture a diverse range of experiences across settings, aiming to identify shared meanings that could reveal core concepts [28].

Department managers were contacted via telephone and email and provided with study information. They, or their designees, recruited participants and shared contact details of those who expressed interest in participating. To increase variation in gender and professional background, two participants were recruited through snowball sampling [28].

Recruitment was initiated without a predetermined sample size and was guided by the concept of information power [31]. Participant characteristics, as well as the richness and relevance of interview data, were assessed after each interview. Recruitment continued alongside interviews until sufficient information power was achieved [31]. Recruitment and data collection were conducted from December 2024 to February 2025.

### Participants

Fifteen participants were invited to the study: ten from in-hospital settings and five from pre-hospital settings. Participants’ demographics are shown in Table 1.

**Table 1:** Participants demographics

Gender	Female (male)	7 (8)
Age (years)	30–39 years	10
	40–49 years	2
	50–59 years	3
	Median, all participants	37
Profession	Paramedic	4
	Emergency medical technician	1
	Physician	2
	Nurse	8
Additional academic education	Paramedic bachelor	1
	Professional Diploma in Health Sciences	1
	Emergency Nursing	2
	Critical care nursing	2
	Cardiac nursing	1
	Cardiac sonography	1
	Mental Health	1
Clinical experience (years)	Paramedics	14–33
	Emergency medical technician	10
	Physicians	2–7
	Registered nurses	5–34
	Median, all participants	10
Annual participation in resuscitation	0–5	4
	5–10	6
	10–15	2
	15–20	3

Organization of the cardiac arrest team varied across hospitals. Participants could serve in different functions across resuscitation attempts, with corresponding changes in responsibility. Participants from in-hospital settings were recruited from the emergency department, cardiac critical care unit, or anesthesiology department. They included nurses, specialized nurses, or anesthesiologists in their first two years of residency. All participants regularly took part in resuscitation efforts. Four participants were certified ALS instructors.

All hospitals had access to CCFDs. Seven in-hospital participants served on their hospital’s cardiac arrest team. In one in-hospital setting, participants served as CPR coaches. Their tasks included supervising chest compression quality, organizing changeovers, selecting the appropriate chest compression modality (e.g., manual or mechanical), and occasionally performing compressions. At another in-hospital setting, participants from the emergency department performed chest compressions, administered medication, and carried out other resuscitation-related tasks. The same participants also routinely served on the hospital’s cardiac arrest team operating the defibrillator unit, timed the ALS algorithm, and documented the resuscitative effort. Participants from the cardiac critical care unit performed similar tasks, including chest compressions, medication administration, and operating the defibrillator unit.

Two hospitals provided staff with structured training on device before implementation, while the third hospital implemented the device without any structured training.

In the pre-hospital setting, four participants were paramedics, and one was an emergency medical technician. Paramedics staffed either a solo paramedic vehicle, an ambulance, or assisted in an emergency physician vehicle. The emergency medical technician was assigned to an ambulance.

The pre-hospital cardiac arrest team followed the Danish pit-crew model [32], which provides a structured framework for team function during resuscitation. The model defines four different zones relative to the patient, with each zone assigned specific ALS tasks. Staffing of zones varied according to profession, as shown in Table 2 [32].

**Table 2:** Role allocation and task distribution in the pre-hospital setting

Zone	Professions	Tasks
1	ALL	Chest compressions
2	EMT Paramedic Physician	Operate defibrillator unit Airway management and ventilation Timing Rhythm and pulse check
3	Paramedic Physician	Intravenous/Intraosseous access Administer medication Team leader (if only three zones are active) Operate mechanical compression device
4	Paramedic Physician	Team leader Medical history Clinical decision-making Triage and dispatch

Personnel in zones one to three and all equipment have predefined positions relative to the patient [32]. The physician in zone four may position themselves freely based on clinical judgment. They may even briefly leave the resuscitation to make phone calls or talk to relatives [32]. When zone four was unmanned, the paramedic in zone three assumed the role of team leader.

### Chest compression feedback devices used by participants

**Fig. 2:**
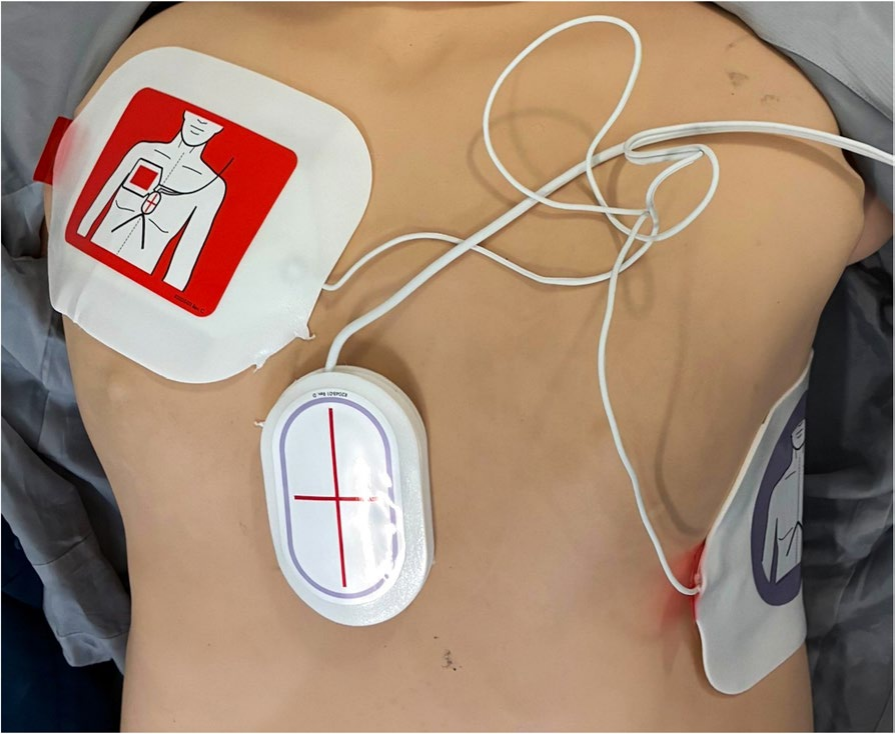
ZOLL’s CPR Stat-Padz Electrodes™ with the CPR sensor separated from the defibrillator electrode

**Fig. 3:**
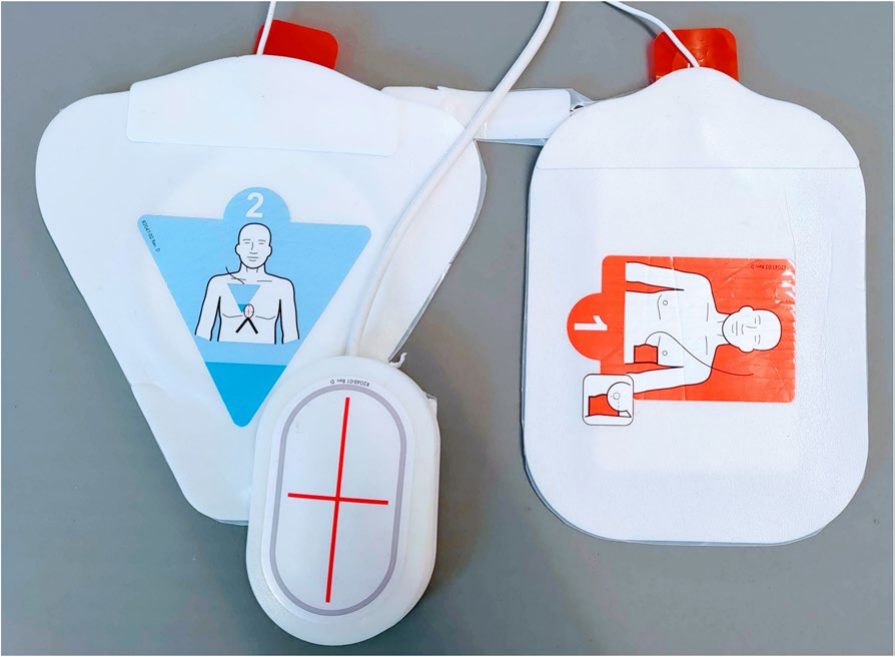
ZOLL’s OneStep™ CPR defibrillator electrodes with the CPR sensor not separated from defibrillator electrode

The type of CCFDs used by the participants varied between hospitals. All CCFDs were integrated into the defibrillator unit. Pre-hospital participants used the ZOLL X Series® defibrillator with ZOLL’s CPR Stat-Padz Electrodes™ (Fig. 2). In-hospital participants used the ZOLL R Series® defibrillator with ZOLL’s OneStep™ CPR defibrillator electrodes (Fig. 3) (ZOLL Medical Corporation, Chelmsford, MA, USA). Both electrode types included a CPR sensor integrated into the anterior defibrillator electrode.

Feedback systems were implemented three to five years prior to data collection. The defibrillator displayed visual feedback on the screen and provided audio feedback through an internal speaker. Feedback modes and volume were preset and not adjustable during resuscitation. The feedback modality varied between the three hospitals, ranging from visual feedback only, to visual feedback combined with an audio metronome, and to visual feedback combined with an audio metronome and voice prompts.

### Data collection

A semi-structured interview guide (Additional file 2) was developed based on prior knowledge and experience, focusing on the device, clinical setting, and organizational factors. The guide included open-ended questions to facilitate dialogue and encourage participants to provide rich descriptions of their experiences. While key topics were covered, the guide remained flexible to allow new topics to emerge [28]. We pilot-tested the interview guide and made minor revisions. Data from the pilot interview were not included in the final dataset.

ØMH conducted all interviews between December 2024 and February 2025 via Microsoft Teams [33], allowing participants to choose the time and location. Each participant completed one interview, and no one withdrew from the study. Participants engaged readily with the topic and openly shared their experiences. RS, who is an experienced qualitative researcher, participated in the pilot interview and two study interviews as co-moderator.

We audio recorded all interviews, totaling 631 min (median: 42 min). Recordings were transferred and stored at Nettskjema [34], which automatically transcribes uploaded recordings using artificial intelligence. Due to technical issues, one interview was recorded separately and uploaded manually. ØMH reviewed all transcripts twice while listening to the recordings, ensuring familiarization with the data, correcting any transcription errors, and de-identifying transcripts. The final dataset comprised 90,589 words. All members of the research team had full access to the de-identified data to facilitate collaborative analysis.

### Data analysis

Data were analyzed using reflexive thematic analysis by Braun and Clarke [35], supported by NVivo 15 [36], where we identified patterns of meaning throughout the dataset. The method aligns with a pragmatic scientific philosophy. It acknowledges the researcher’s active role in constructing knowledge through reflexive and iterative engagement with the data [29, 35]. The analysis followed a non-linear process in which insights from one phase often led to revisiting earlier phases for re-analysis.

ØMH was responsible for the main body of the analysis and conducted phase one (familiarization) and phase two (coding). Inductive coding was applied to both semantic and latent content, while engaging in regular reflexive dialogue with RS. In phase three (generating initial themes), ØMH constructed preliminary themes (Table 3). ØMH, CBI, and RS reviewed the themes, followed by reflexive discussions. Themes were reviewed for internal consistency and external distinction during phases four and five (reviewing themes and defining and naming themes). All researchers (ØMH, CBI, BSS, RS) participated in phase six (writing).

**Table 3:** Examples from the analysis process for the theme: Feedback—device-to-rescuer communication

Representative quote	Code	Theme
…it just keeps talking. It's like that aunt who never gets tired of reminding you to keep going.”	A loud disturbing sound	Feedback: Device-to-rescuer communication
“The feedback volume was hysterically loud—like level 10. It really stressed people out. It doesn’t matter if you see the value in using the device; it’s not a useful tool if it hurts your ears.”		
“…they (the compressor) often get tunnel vision. So, I don’t think everyone perceives what it’s (the defibrillator) saying.”	Compressors struggle to perceive audio feedback	
“I find it difficult to hear all the feedback when I’m performing compressions. That’s because there are often several things happening at once.”		
“It should be around 110, and you can see how deep you’re compressing here.”	Visual feedback is easy to follow	
“Okay, great, now I can… now I can see what I’m doing, and what I need to do.”		

Scientific curiosity and reflexivity guided the analytical process. ØMH is a critical care nurse and certified ALS instructor. CBI is an anesthesiologist, cardiologist, and researcher. BSS is an anesthesiologist and quantitative researcher. RS is a critical care nurse and qualitative researcher. All have experience in resuscitation. Their varied backgrounds allowed the researchers to take different positions on the phenomenon, which fostered fruitful discussions and insights.

## Results

Based on a reflexive thematic analysis, we constructed four themes that captured healthcare professionals’ experience with enablers and barriers to the use of CCFDs: *(1) The CPR sensor—a key physical interaction point; (2) Feedback—device-to-rescuer communication; (3) Organizing cardiac arrest treatment; and (4) Perceived usefulness—an important enabling factor.*

### The CPR sensor—a key physical interaction point

As the only physical interaction between the rescuer and the device, participants’ experiences with the CPR sensor significantly shaped their overall views of the CCFD. The sensor was described as prone to shifting positions during the resuscitation attempt, particularly in diaphoretic patients, or due to difficulty applying vertical compression forces:

*“… in real-life situations, you often find yourself in uncomfortable positions—like not being able to get properly on top of the patient. During VT or a cardiac arrest, patients are often diaphoretic. Even if you wipe the chest with alcohol, the moisture tends to return. Then it feels like the sensor tends to shift, especially if you're leaning over.” (Participant 1)*

The manufacturer provided the CPR sensor together with the defibrillator electrodes, which was considered essential for ensuring its use in resuscitation. The need to perform several interventions rapidly and simultaneously often influenced sensor placement. A common practice was to place the anterior electrode before the CPR sensor to comply with guidelines emphasizing early defibrillation. In patients whose chest anatomy did not align with electrode design, sensors could end up too high, low, or lateral. Although the sensor is integrated into the defibrillator electrode, it can be separated, but many considered this time-consuming and did not prioritize it. Participants occasionally observed incorrectly positioned CPR sensors, yet placement was rarely verified after application. Repositioning the sensor reduced its adhesive qualities. Notably, one department prioritized sensor placement, sometimes resulting in electrodes being placed several centimeters below the clavicle. Misplaced or loose sensors caused frustration and were often removed, rendering the CCFD unusable.

Most participants found compressing on the CPR sensor more comfortable. The sensor is slightly cushioned, reduces the angle between the hand and the chest, and supports correct hand placement. Experiencing the patient’s ribs and sternum fracturing was common, and in some cases, the skin on the patient’s chest peeled off during compressions. Some participants described this as emotionally challenging and transgressive, particularly when compressing directly on the chest. The CPR sensor served as a physical buffer, creating an emotional distance that helped participants focus on performing chest compressions.

Participant 13 said:

“*Even after all these years, I still find it deeply unpleasant to compress directly on a patient’s chest. It feels transgressive, and it’s something I’ve never fully gotten used to. Sometimes you see how the skin rolls off, and in a way, it's not nearly as invasive when you have the puck in between. … It’s entirely possible that I have applied less force because I felt uncomfortable.”*

### Feedback—device-to-rescuer communication

Participants described how rescuers often developed tunnel vision when performing chest compressions, reducing their ability to receive audio feedback. Although one participant deliberately disregarded visual feedback, it was generally considered informative and easy to follow, even for bystanders:

*“… when the defibrillator says, for example, if it says ‘press harder*’ *or something like that, I  felt like they didn’t catch it. And when there's a frequency error—compressions outside the guideline rate—it’s just a kind of beeping and buzzing. So, I experienced that they don't catch it, and I must guide them."* (*Participant* 4)

Rescuers performing chest compressions often struggled to perceive audio feedback, whereas other team members heard it clearly and considered it disruptive to the resuscitation attempt. In some cases, this resulted in sensor removal or the use of defibrillator electrodes without a CPR sensor:

*“…the anesthesiologist said: ‘We need to  switch to another defibrillator. This one is driving me crazy.*’ *It was constantly talking, and that's not necessary.”* (*Participant* 8)

Participants from one department reported lowering the feedback volume, which reduced team disruptions and enabled continued use of the device.

### Organizing cardiac arrest treatment

Participants described varying organizational structures of cardiac arrest teams across the three hospitals. In two hospitals, formal protocols and team structure enabled consistent use of CCFDs by assigning responsibility and facilitating ownership.

In these two hospitals, the local cardiac arrest protocol mandated the use of CCFD alongside other available systems for monitoring compression quality. Additionally, the cardiac arrest teams functioned as two distinct sub-teams. One sub-team was led by an experienced physician who assumed overall responsibility for the resuscitation and focused on clinical decision-making. The second team functioned as a CPR sub-team responsible for CPR-related tasks, including chest compressions, ventilation, defibrillation, and medication, and managed the ALS algorithm.

Although not always formally defined in both settings, participants described a recurring pattern where leadership within the CPR sub-team was assumed by the paramedic operating in zone 3 or the anesthesiologist serving as CPR coach. In the in-hospital setting, this division of labor was formalized in the local cardiac arrest protocol. In the pre-hospital setting, it developed informally as a result of organizing the cardiac arrest team according to the pit-crew model. In this model, the paramedic in zone 3 naturally assumed responsibility for the ALS algorithm through a shared understanding with the physician, while the physician in zone 4 primarily focused on clinical decision-making. The protocol also specified a fixed defibrillator placement relative to the patient, ensuring that visual feedback was clearly visible to both the chest compression provider and CPR sub-team leader, supporting real-time assessment of compression quality.

A formal responsibility to ensure high-quality chest compressions and to use the CCFD, combined with a leadership role within the CPR sub-team, fostered a sense of ownership and accountability. This emerged as an important enabler for consistent use. This sense of responsibility was reflected in how CPR sub-team leaders described their role:

*“It's my job to ensure there’s an adequate cardiac output. Broadly speaking, I use two things to assess it: the puck, to evaluate the quality of the compressions, and the end-tidal CO*_*2*_*—the capnography.”* (*Participant* 15)

At the third hospital, the cardiac arrest team operated as a single unit with clearly defined roles. However, no team member was specifically assigned responsibility for chest compression quality. Participants described the treating physician and team leader as ultimately responsible but noted that this person was often occupied with clinical decision-making and therefore unable to assess compressions directly. Consequently, the responsibility for monitoring compression quality was diffuse, and often shared implicitly among team members as participant 2 stated:

*“No, there isn't anyone dedicated to that, no. There is the team leader, of course—the cardiologist. Although, they use their capacity to find reversible causes…”*

Neither did the local cardiac arrest protocol define whether or how the CCFD should be used, nor did it specify a fixed defibrillator placement relative to the patient. As a result, visual feedback was occasionally only visible to the team member operating the defibrillator, leaving the chest compression provider reliant on audio feedback. The lack of formal responsibility sometimes led to the use of defibrillator electrodes without a CPR sensor, and more commonly, audio feedback being broadcast without a clearly defined recipient.

When practical, some participants attempted to place the defibrillator units so that visual feedback was visible to the chest compression provider and team. They also relayed feedback on their own initiative. However, these efforts occurred informally on a person-to-person basis, resulting in inconsistent use.

Participants from the pre-hospital service tended to adhere more closely to institutional procedures, while in-hospital participants relied more on individual judgment and informal routines. This contextual difference may have influenced how consistently the CCFD was used across settings.

### Perceived usefulness—an important enabling factor

Perceived usefulness of the CCFD varied across roles and settings and emerged as a key enabler for its integration into clinical practice. Participants could hold diverse roles across resuscitation attempts, and usefulness depended more on role than on individual. This became particularly evident when comparing participants’ experience as chest compression providers versus CPR sub-team leaders.

When performing chest compressions, participants relied primarily on personal experience and subjective assessment of quality, although some occasionally referred to the defibrillator for confirmation. In contrast, when serving as CPR sub-team leaders, participants emphasized the value of access to objective CPR performance data, which supported more accurate assessment of chest compression quality. The CCFD also enabled additional defibrillator functions, such as filtered electrocardiogram (ECG) and an algorithm timer, which facilitated rapid rhythm assessment and team coordination. One participant placed the sensor on the upper part of the chest, after switching to mechanical compressions to retain these functions.

In the pre-hospital setting, an app-based first responder system resulted in volunteer bystanders attending up to 85% of all cardiac arrests. With no requirement for previous CPR training, first responders varied in both skill level and familiarity with cardiac arrest situations.

Participant 11 said:

*“We said: ‘You can see how deep you are compressing here.*’ *Then I noticed that he was like: Okay, fine, now I can... Now I can see what I'm doing, and what I need to do, and I get feedback*.’ *So, my paramedic colleague and I could then focus on airway management and venous access.”*

Guiding bystanders to follow feedback displayed on the defibrillator screen enabled them to deliver and sustain high-quality chest compressions, thereby positioning them as a valuable resource during resuscitation efforts.

While participants valued access to objective data on CPR metrics, most had experienced being instructed to adjust hand position based on physiological parameters such as end-tidal CO2 or invasive blood pressure, to optimize chest compressions. They reflected that tailoring compressions to individual physiological responses may improve outcomes, and questioned the future role of current CCFDs in such contexts.

## Discussion

Enablers and barriers to device use were identified in how clinicians interacted with the CPR sensor, the nature of feedback, team organization, and the perceived clinical benefit.

### The CPR sensor–usability, comfort, and emotional distance

The CPR sensor’s design and placement influenced both usability and emotional experience, shaping how the device was perceived.

Clinicians have previously described how they occasionally forgot to apply the CPR sensor [18]. In this study, the sensor was integrated into the anterior electrode, which ensured its availability during the resuscitation attempt, reducing the likelihood that it would be forgotten. A key barrier reported by participants was the challenge of correctly positioning and maintaining sensor placement. Participants reported how the CPR sensor was rarely separated from the defibrillator electrode. This reflected a perceived conflict between guideline recommendations for early defibrillation [4] and the perceived time cost of separating the CPR sensor from the defibrillator electrode. As defibrillator electrodes were usually placed before the sensor, the sensor could become misaligned with the recommended compression point. Although previous studies have reported challenges with sensor placement [37, 38], none of the hospitals had routines for verifying its position, as this was not specified in their local cardiac arrest protocols. This lack of procedural guidance may hinder the use of CCFDs and contribute to inconsistent application.

Participants described how attachment and repositioning difficulties led to frustration and a negative attitude toward the system, effectively acting as a barrier to the use of CCFDs. Loose sensors were often removed during resuscitation attempts, rendering the device ineffective.

The dilemma of when to apply the sensor has been previously described [19], with prioritizing other interventions before placing the sensor often leading to the CCFD not being used. This uncertainty acts as a barrier to the use of CCFDs and highlights the need for local protocols to define the sequence of equipment application. Without such guidance, CCFDs may be deprioritized despite their potential to improve chest compression quality [12, 14, 15, 17]. In one department, prioritizing sensor placement occasionally led to suboptimal electrode positioning, illustrating how competing priorities and workflow can discourage correct sensor placement.

Clinicians described the sensor as comfortable, highlighting its cushioning and support for correct hand placement. Their experience was generally positive, provided the sensor remained securely in place. Unlike earlier studies [19, 21, 39], participants reported no discomfort when performing compressions on the sensor. This likely reflecting device heterogeneity [13] and improvements in design, highlighting the importance of user-centered design. Importantly, comfort extends beyond physical ergonomics. As described in this and previous studies [7, 40], feeling emotional discomfort when compressing directly on the patient’s chest is not uncommon. Participants described how the CPR sensor created physical and emotional distance, helping them maintain focus and reducing the sense of personal intrusion. This dual role—providing both physical and emotional comfort—was perceived as an enabler for device use, a factor not been described in previous research. These findings suggest that emotional ergonomics may be an overlooked factor in device design and training protocols.

### Feedback as an enabler and barrier

This study found that feedback from the CCFD functioned both as an enabler and a barrier to device use. In line with a previous study [24], participants described audio feedback as particularly challenging. Performing chest compressions requires a high level of cognitive attention [8], and participants noted that chest compression providers often developed tunnel vision, which limited their ability to perceive auditory signals. In addition, audio feedback was reported to cause alarm fatigue, interrupt team communication and divert focus from critical tasks. In previous studies, clinicians muted audio feedback in 14–18% of cases [15, 26]. In contrast, muting audio feedback was not possible for participants in this study. Participants described how audio feedback could disturb resuscitation attempts, sometimes leading to sensor removal or the use of electrodes without the CPR sensor, rendering the feedback system non-functional.

Visual feedback, on the other hand, was considered easy to follow and did not disturb team communication; it was also the preferred modality. While disabling audio feedback may seem reasonable to reduce disruptions, it creates a dilemma: the system’s ability to alert the team to poor compression quality may be diminished. Evidence indicates that audio-visual feedback is more effective than visual feedback alone in improving patient-centered outcomes [17], suggesting that removing audio may reduce the clinical benefit of the device.

Participants reported relying more on own experience when performing compressions than when supervising others. In contrast, the participants both valued and relied on real-time feedback when supervising chest compressions and appreciated its ability to guide inexperienced chest compression providers. This raises an important question: *to whom should feedback be directed*? Previous research indicates that feedback must be clear and easily perceivable to be accepted by rescuers [24]. Difficulties faced by chest compression providers in perceiving audio feedback, as well as differences in perceived usability of real-time feedback, suggest that feedback should be tailored in both modality and direction.

Since visual feedback is considered easy to follow, and does not disrupt resuscitation, visual feedback should be directed towards the chest compression provider. Further, as participants both valued and relied on real-time feedback when supervising chest compressions there is a need for a method for alerting the sub-team leader if compressions fall below guideline standards, preferably without disturbing team communication.

To accommodate these differing needs and minimize disruption to team communication, one potential solution is the use of haptic feedback in combination with visual feedback.

For example, vibration [41] could be delivered through a wireless wearable device, such as a wristband or armband, to the sub-team leader. This discreet alert would draw attention to poor compression quality without interrupting team communication, allowing timely intervention. Due to tunnel vision and high cognitive load [8], chest compression providers should continue to rely on visual feedback to maintain guideline-adherent compressions.

### Organizing cardiac arrest teams – team structure and equipment setup

The organization of cardiac arrest teams and assignment of responsibility significantly influenced how CCFDs were perceived and used.

Two hospitals practiced a predefined equipment layout, ensuring that visual feedback was always visible—an essential condition for the use of CCFD. One hospital had a CPR coach responsible for supervising chest compression quality and coordinating CPR-related tasks. Being responsible for this coordination, CPR coaches often assume informal leadership over those directly involved in performing CPR [42]. This creates a division of the cardiac arrest team into two sub-teams [42], a pattern also recognizable in this study. In the pre-hospital setting, the cardiac arrest team was organized according to the pit-crew model (Table 2) [32]. The paramedic in zone 3 often assumed leadership over individuals operating in zone 1 and zone 2, based on a shared understanding with the leading physician in zone 4. This leadership role included supervising chest compression performance and effectively serving as a CPR coach. Because subjectively assessing chest compression quality is challenging [10], this responsibility created a need for objective feedback. This need prompted intended use of the CCFD and fostered a sense of ownership of the device, ensuring consistent utilization.

In contrast, in the hospital where no team member was designated to supervise quality, participants described this responsibility as shared and diffuse. This lack of clear responsibility also resulted in a lack of ownership of the device, meaning no one felt accountable for its use. Therefore, the defibrillator was sometimes placed such that visual feedback was obstructed, audio feedback was broadcast without a designated receiver, or the sensor was not applied at all. The absence of ownership led to inconsistent use or complete omission of the device, creating a barrier to its integration into practice.

These experiences suggest that usability is shaped not only by the device design or feedback modality. It also depends on how the device is embedded in structures and supported by clear role assignment. While the CPR coach role may arise informally [42], formalizing it clarifies the responsibility for supervising chest compression quality. This appears to be a strong enabler for consistent and effective use of the CCFD, as it clarifies accountability and supports the need for objective CPR performance data.

### Perceived usefulness

In ALS, delivering high-quality chest compressions is a top priority [4], yet it remains challenging [11, 12]. Performing compressions may be perceived as physically, cognitively, and emotionally demanding [7–9, 39]. Given that the aim of CCFD is to enhance chest compression performance [13], it is reasonable to assume that chest compression providers value real-time feedback to improve quality [12, 14, 15, 17]. However, participants described role-based differences in perceived usefulness. They noted that real-time feedback was valued more when holding supervisory roles than when performing compressions. This may be explained by the challenges associated with subjectively assessing chest compression quality [10], and the responsibility to oversee all aspects of CPR performance [42]. In contrast, chest compression providers may experience increased fatigue [39] and cognitive load when using a CCFD [9] which may explain why the device is less valued when performing chest compressions.

To function, the CCFD requires the chest compression provider to perform compressions directly on the CPR sensor. This creates a situation where the benefits of sensor use—such as real-time feedback, filtered ECG, and algorithm timer—are distributed across the resuscitation team, while the burden of use is concentrated on the chest compression provider. Therefore, assigning responsibility for device utilization to the sub-team leader may be reasonable, as they derive the greatest benefit from its functions.

While prior studies have highlighted familiarity with the device as an important enabler for acceptance [23, 24], this was not a consistent theme in our data. Out-of-hospital cardiac arrest is more common than in-hospital [1], and pre-hospital participants tended to adhere more closely to institutional procedures than in-hospital participants. This suggests that contextual factors—such as team structure, procedural adherence, and familiarity—should be considered key when designing implementation strategies for CCFDs. These factors are also important when determining who should be responsible for utilizing the device.

### Future development of chest compression feedback devices

CCFD’s ability to improve patient-centered outcomes has previously been identified as vital for adoption [24]. Most participants were familiar with the use of end-tidal CO_2_ to confirm tube placement and assess for return of spontaneous circulation. Although participants valued access to objective CPR metrics, they noted that current devices do not support physiological feedback—such as end-tidal CO_2_, blood pressure and flow readings—which limited their ability to individualize chest compressions. While end-tidal CO_2_ is used as an indirect marker of circulation, it is a secondary measure and does not provide direct feedback on compression effectiveness [3, 13].

Beyond EtCO_2_, a few participants described being guided by physicians using invasive blood pressure monitoring to optimize compression point, illustrating clinicians’ reliance on physiological indicators when available. Emerging technologies for assessing the effect of chest compressions such as quantitative pupillometry and hands-free Doppler measurement of carotid blood flow have been suggested in research. These approaches are considered promising but remain at an experimental stage [43, 44].

Participants considered the use of physiological feedback an opportunity for improving patient-centered outcomes. Current CCFDs are designed to remain in a fixed position during resuscitation [13]. Repositioning the sensor compromises its adhesive properties, making it unsuitable for dynamic adjustment of the compression point. This limitation reduces the potential for individualizing chest compressions when using CCFDs. As clinicians increasingly adopt physiological data to guide resuscitation [44–46], participants considered future CCFDs should be redesigned to integrate such feedback, thereby supporting more informed clinical decision-making during CPR. While the current CCFDs provide feedback on the performance of chest compressions (for example, rate, depth), physiological parameters such as EtCO_2_, intra-arterial blood pressure, and potentially quantitative pupillometry or Doppler flow measurement offer information on the effect of compressions on circulation and perfusion. These measures of blood flow provide feedback on the effect of chest compressions and should complement—not replace—real-time mechanical feedback to support both individualization and consistency in CPR quality [24, 45]. Incorporating physiological feedback into future designs to enable dynamic adjustment of compression depth and point may represent a key enabler for the clinical use of CCFDs. This approach could enhance clinical utility by enabling individualized CPR and supporting patient-centered outcomes.

### Mechanical chest compression devices: implications for CCFDs

Mechanical chest compression devices were not included in the study framework as our focus was manual compression with CCFDs. Guidelines recommend mechanical devices primarily during prolonged resuscitation attempts, patient transportation, or when personnel are limited [4, 5]. Manual compressions remain the standard approach in most settings [4, 5], which underscores the continued relevance of CCFDs for optimizing CPR quality.

A few participants briefly discussed their use of mechanical chest compression devices in resuscitation and reported using mechanical compressions only when manpower was insufficient or during transport. One highly experienced participant described starting with manual compressions and moving the sensor around the patient’s chest to identify the position yielding the highest EtCO_2_ value and then mounting the mechanical device’s piston at that location. Although repositioning the sensor compromised its adhesive properties, its use was brief and primarily aimed at optimizing piston placement before initiating mechanical compressions.

Currently, both CCFDs and mechanical devices lack the ability to provide feedback on the physiological effect [47]. Further research should explore technologies that provide physiological feedback for both manual and mechanical compressions [47].

### Strengths and limitations

A strength of the study lies in its adherence to Lincoln and Guba’s criteria to ensure trustworthiness [28], and its use of the COREQ checklist [30], which enhances transparency and rigor. All participating hospitals used defibrillators, electrodes, and CPR sensors from the same manufacturer, which may limit transferability to settings using other equipment.

Participants represented both Norwegian and Danish ALS settings. Although the healthcare systems in these countries are generally comparable, differences in organizational structures, training practices, and implementation strategies may influence how the findings are recognized and applied in other settings. Nevertheless, consistent accounts for participants across these contexts strengthen the credibility of the findings and suggest potential transferability to other ALS environments [28].

Participants held diverse professional and clinical backgrounds, yet all were experienced ALS providers. This contributed to rich and nuanced descriptions of their experiences with CCFDs. The use of digital interviews limited our ability to read body language. However, using this format allowed participants to choose time and setting for the interview, and created a distance that may have increased their comfort and openness [48]. Digital communication is increasingly common in qualitative research and its use is considered methodologically acceptable [48].

The research team consisted of four authors with differing backgrounds, which enabled us to approach the phenomenon differently and fostered critical discussions through the process. In line with reflexive thematic analysis [35], transcripts and analyzed material were not returned to the participants, as the method emphasizes the researcher’s interpretive role rather than participant validation.

## Conclusions

This study identified key enablers and barriers to the use of chest compression feedback devices in advanced life support. Usability of the CPR sensor, clarity of feedback, and team organization emerged as critical factors influencing both implementation and sustained use. While sensor ergonomics generally supported adoption, challenges related to positioning and detachment often led to sensor removal or discontinued use. Visual feedback was consistently preferred over audio feedback, which was frequently perceived as disruptive. Real-time feedback was particularly valued in supervisory roles, compared to when performing chest compressions. Furthermore, formal assignment of responsibility, combined with integration into cardiac arrest protocols, promoted consistent utilization. Overall, these findings suggest that successful implementation of chest compression feedback devices requires user-centered design, role-sensitive feedback modalities, and context-specific protocols. Further development should focus on providing multimodal feedback tailored to distinct team roles and integrating physiology-guided resuscitation strategies to enhance clinical utility and relevance.

## Supplementary Information

Additional file 1.

Additional file 2.

## Data Availability

The datasets used and/or analyzed during the current study are available from the corresponding author on reasonable request.

## Abbreviations

ALS: Advanced life support
CCFD: Chest compression feedback device
COREQ: Consolidated criteria for reporting qualitative research (COREQ)
CPR: Cardiopulmonary resuscitation
ECG: Electrocardiogram
