# Supplementary material for: Exploring enablers and barriers to the use of chest compression feedback devices in advanced life support: a qualitative study
Source: Scand J Trauma Resusc Emerg Med. 2026 Apr 1;34:96. doi: 10.1186/s13049-026-01607-3 (PMC13217654; doi:10.1186/s13049-026-01607-3)
Supplement: Supplementary file 2 — Additional file 2. [file 13049_2026_1607_MOESM2_ESM.pdf]

Interview guide

Themes	Main questions	Follow-up questions
Introduction	**Can you describe a situation where you were involved in a cardiac arrest and the chest compression feedback device was used?**	• What are your experiences using a chest compression feedback device<br>• Describe how you think the device contribute to the resuscitation effort
The cardiac arrest organization	**Can you describe how the chest compression feedback device is intended to be used in your hospital?**	• Are there any formal or informal guidelines for using the chest compression feedback device. If so, what are they, and are they easily accessible.<br>• Do you use the feedback device in all arrests? If not, when and why?<br>• Do you use the device in ALS training? If so, why/why not, and how often?
The device	**How did it feel to use the feedback device during CPR?**	• Tell me about the CPR sensor<br>• Are there any challenges? Do you find the device reliable?<br>• Have you used other feedback devices
The cardiac arrest situation	**In what ways does the feedback device influence the quality of resuscitation, team dynamics, or decision-making during CPR?**	• How do you experience feedback?<br>• Do you consider feedback helpful.<br>• Do you prefer visual or audial feedback. Please elaborate.<br>• Do you consider the feedback device valuable for assessing chest compression quality.
Enablers and barriers	**Can you describe any conditions or practices at your workplace that make it easier to use the feedback device during resuscitation**	
Is the anything you want to add, comment on, just talk about?	**General follow-up questions:**<br>- Please elaborate. Why is it so?<br>- Please explain more closely.<br>- Have you more/other examples?<br>- Can you go further into details?<br>- What do you think?	
